# Serum Soluble Triggering Receptor Expressed on Myeloid Cells-1 and Procalcitonin Can Reflect Sepsis Severity and Predict Prognosis: A Prospective Cohort Study

**DOI:** 10.1155/2014/641039

**Published:** 2014-02-04

**Authors:** Zhenyu Li, Hongxia Wang, Jian Liu, Bing Chen, Guangping Li

**Affiliations:** ^1^Intensive Care Unit, The Second Hospital, Tianjin Medical University, Tianjin 300211, China; ^2^Cardiology Department, The Second Hospital, Tianjin Medical University, Tianjin 300211, China

## Abstract

*Objective*. To investigate the prognostic significance of serum soluble triggering receptor expressed on myeloid cells-1 (sTREM-1), procalcitonin (PCT), N-terminal probrain natriuretic peptide (NT-pro-BNP), C-reactive protein (CRP), cytokines, and clinical severity scores in patients with sepsis. *Methods*. A total of 102 patients with sepsis were divided into survival group (*n* = 60) and nonsurvival group (*n* = 42) based on 28-day mortality. Serum levels of biomarkers and cytokines were measured on days 1, 3, and 5 after admission to an ICU, meanwhile the acute physiology and chronic health evaluation II (APACHE II) and sequential organ failure assessment (SOFA) scores were calculated. *Results*. Serum sTREM-1, PCT, and IL-6 levels of patients in the nonsurvival group were significantly higher than those in the survival group on day 1 (*P* < 0.01). The area under a ROC curve for the prediction of 28 day mortality was 0.792 for PCT, 0.856 for sTREM-1, 0.953 for SOFA score, and 0.923 for APACHE II score. Multivariate logistic analysis showed that serum baseline sTREM-1 PCT levels and SOFA score were the independent predictors of 28-day mortality. Serum PCT, sTREM-1, and IL-6 levels showed a decrease trend over time in the survival group (*P* < 0.05). Serum NT-pro-BNP levels showed the predictive utility from days 3 and 5 (*P* < 0.05). *Conclusion*. In summary, elevated serum sTREM-1 and PCT levels provide superior prognostic accuracy to other biomarkers. Combination of serum sTREM-1 and PCT levels and SOFA score can offer the best powerful prognostic utility for sepsis mortality.

## 1. Introduction

Sepsis is the major cause of death in the intensive care unit. Despite improvement of antibiotics treatment and supportive techniques, the mortality of septic shock increases to approximately 60% [1]. Recently biomarkers are widely used to diagnose and manage sepsis. As a good biomarker, it not only helps doctors to make an early diagnosis of sepsis, but also predicts outcomes. Meanwhile, it should be easily available and cost cheap.

There have been some biomarkers and cytokines used in both the clinical practice and laboratory including soluble triggering receptor expressed on myeloid cells-1 (strem-1), procalcitonin (PCT), N-terminal probrain natriuretic peptide (NT-pro-BNP), C-reactive protein (CRP), interleukin-6 (IL-6), and interleukin-10 (IL-10). TREM-1 is a recently discovered member of the immunoglobulin superfamily of receptors that is specifically expressed on the surfaces of neutrophils and monocytes [2]. sTREM-1 is a soluble form of TREM-1 and is upregulated when exposed to infectious diseases [3]. PCT is a polypeptide consisting of 116 amino acids and is the precursor of calcitonin; it was proven useful to identify nonsystemic inflammatory response syndrome and was firstly used in sepsis [4]. NT-pro-BNP is a biologically inactive form that is cleaved from the prohormone probrain natriuretic peptide (pro-BNP) by proteolytic enzymes before secretion [5]. CRP is a widely used biomarker to discriminate the inflammatory response to sepsis [6]. IL-6 and IL-10 are important proinflammatory and anti-inflammatory cytokines during sepsis course.

Many studies have compared the diagnostic value of biomarkers for sepsis. Gibot et al. [7] indicated that sTREM-1 was more accurate than PCT and CRP in the diagnosis of sepsis, but others showed that the prognostic utility of serum sTREM-1 in septic shock was inferior to that of PCT [8]. The prognostic value of biomarkers mentioned above for sepsis is unclear. The purpose of the study was to compare the prognostic value of biomarkers and cytokines versus clinical severity scores and improved death risk prediction.

## 2. Materials and Methods

### 2.1. Study Population

A total of 102 patients with sepsis from single centre hospital intensive care unit were enrolled from December 2010 to August 2012 according to the 2001 International Sepsis Definition conference [9]. The patients were divided into survival group and nonsurvival group based on 28-day mortality. Exclusion criteria included: age younger than 18 years, preexisting thyroid disease and lung cancer that influence procalcitonin levels, patients with acute coronary syndromes and renal dysfunction, and patients staying in ICU less than 24 hours. The study was approved by the hospital's ethics committee and either the patients or their relatives provided informed consent.

### 2.2. Data Collection

Demographic and disease data of patients included age, gender, chief complaints for admission, vital signs, length of stay in ICU, infection sites, microorganisms, routine blood test results, liver and kidney functions, coagulation indicators, blood gas analysis, acute physiologic assessment and chronic health evaluation (APACHE) II scores, and sequential organ failure assessment (SOFA) scores. These were recorded on 3 days (days 1, 3, and 5). Serum was collected at these same time points and PCT, sTREM-1, NT-pro-BNP, CRP, IL-6, and IL-10 levels were determined in the end.

### 2.3. Assay

PCT was measured using an enzyme-linked fluorescence analysis kit (ELFA, VIDAS BRAHMS PCT kit, bioMerieux SA, France). sTREM-1 was determined using a double antibody sandwich ELISA (Quantikine Human TREM-1 Immunoassay ELISA Kit, R & D Systems, Minneapolis, MN, USA). NT-pro-BNP was measured with an available immunoassay analyzer (Elecsys 2010; Roche Diagnostics, Mannheim, Germany). CRP was determined using scattering using a nephelometric assay (Dade-Behring, SA Paris, France). IL-6 and IL-10 were determined using ELISA (IMMULITE; Diagnostics Products Corporation, Los Angeles, CA). All assays were performed according to the manufacturer's instructions.

### 2.4. Statistical Analysis

Quantitative data with normal distributions are given as means ± standard deviations (SD). Student's *t*-test was used to compare means between two groups. Quantitative data that were not normally distributed were summarized as medians (interquartile ranges) and compared by nonparametric tests (Mann-Whitney *U* test). We made a logarithmic conversion for the nondistribution data when we did dynamic comparison in Figure 2. Proportions were used to express qualitative data and the differences in proportions between groups were compared using a chi-square test. We compared the characteristics of survivors versus nonsurvivors using univariate analysis and used receiver operating characteristics (ROC) curves to evaluate prognostic value of the biomarkers and cytokines predicted 28-day mortality. Those variables with *P* values less than 0.05 on univariate analysis were then entered into a multivariate logistic regression analysis to further identify the independent predictors of 28-day mortality. A *P* value less than 0.05 was considered significant. All tests were two-tailed. Statistical analysis used SPSS Statistics 16.0 and GraphPad Prism 4.0 softwares.

## 3. Result

### 3.1. Patients Characteristics

A total of 102 patients with sepsis were included in this study. The 28-day mortality rate was 41.2%. The mean patient age was 63 ± 21 years. There were no significant difference in age and sex of these two groups (*P* > 0.05). The APACHE II and SOFA scores of patients in the nonsurvival group were higher than those of patients in the survival group (*P* = 0.000, *P* = 0.000, resp.), (Table 1).

### 3.2. Comparison of Serum Biomarkers and Cytokines Levels on Day 1

Serum PCT, strem-1, IL-6 levels of patients in the nonsurvival group were significantly higher than those in the survival group on day 1 (*P* < 0.001). There were no differences in NT-pro-BNP and CRP, IL-10 levels between the two groups (*P* > 0.05) (Table 2).

### 3.3. Univariate Predictors of 28-Day Mortality on Day 1 Based on ROC

**Figure 1:**
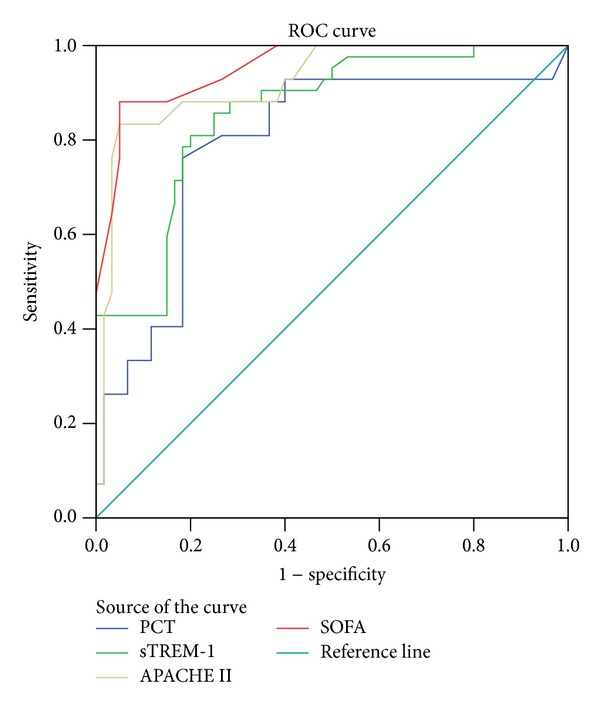
ROC curves for PCT, sTREM-1, SOFA, and APACHEII scores for predicting 28-day mortality on day 1. The area under a ROC curve for the prediction of 28-day mortality was 0.792 for PCT, 0.856 for sTREM-1, 0.953 for SOFA score, and 0.923 for APACHEII score. PCT and sTREM-1 showed the equal prognostic values which were inferior to SOFA and APACHEII scores (*P* < 0.05).

The ROC analysis showed that the accuracy of the PCT, strem-1, IL-6, APACHEII, and SOFA scores on day 1 for the prediction of 28-day mortality was moderate (AUC > 0.7, *P* < 0.01), whereas the accuracy of NT-pro-BNP, CRP, and IL-10 was low (AUC < 0.7, *P* > 0.05) (Figure 1). Comparing AUC of PCT, strem-1, and IL-6, we found that there was no significant difference of AUC between strem-1 and PCT (*P* = 0.2910), and the AUC of the two markers were higher than that of IL-6 (*P* > 0.05). Meanwhile, there was no significant difference of AUC between APACHEII and SOFA scores (*P* = 0.3753). The AUC of APACHEII and SOFA scores were higher than those of strem-1 and PCT (*P* < 0.05) (Table 3).

### 3.4. Multivariate Comparisons of Biomarkers with Cytokines and Clinical Severity Scores

The baseline day 1 variables that were found to be significantly different between survivors and nonsurvivors on univariate analysis (PCT, strem-1, IL-6, APACHEII, and SOFA scores) were entered into a logistic regression model. Among these variables, three variables remained independently associated with 28-day mortality: strem-1, PCT, and SOFA score (Table 4).

### 3.5. Dynamic Changes of Biomarkers and Cytokines Levels

**Figure 2:**
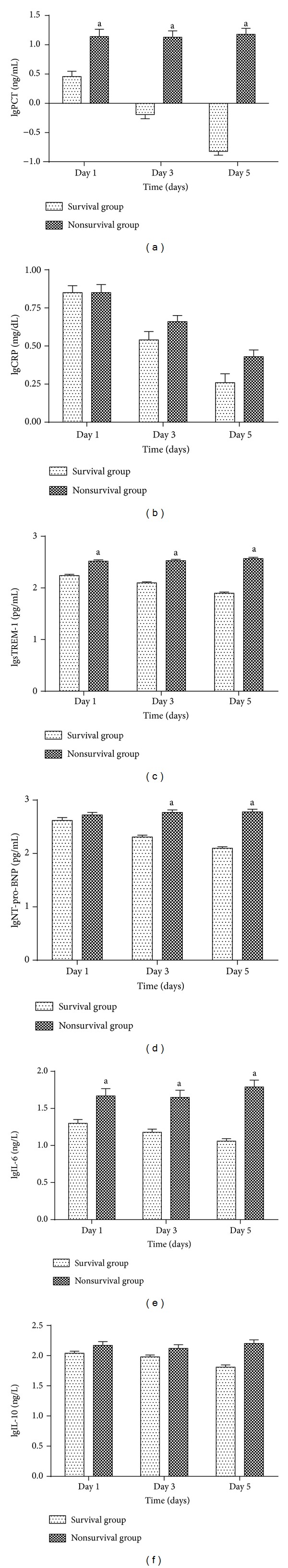
Dynamic changes of biomarkers and cytokines levels in survival group and nonsurvival group. Non-normally distribution data were made a logarithmic conversion into normally distribution data and expressed as means ± standard deviations (SD). ^a^ *P* < 0.01 for comparisons between two groups on the same day (Student's *t*-test).

Median serum biomarkers and cytokines levels were determined on days 1, 3, and 5 and were compared between the survival and nonsurvival groups. Serum PCT, strem-1, and IL-6 levels in the nonsurvival group were higher than those in the survival group on days 1, 3, and 5 (*P* < 0.01). There was no difference in NT-pro-BNP levels on day 1 (*P* > 0.05), but later the NT-pro-BNP levels in the nonsurvival group were higher than those in the survival group on days 3 and 5 (*P* < 0.05). There were no differences in CRP and IL-10 levels on days 1, 3, and 5. Serum PCT, strem-1, IL-6, and NT-pro-BNP levels showed a decrease trend in the survival group (*P* < 0.05), but there was no decrease tendency in the nonsurviving group for these four biomarkers; strem-1 even had a increase trend (*P* < 0.05). Serum CRP levels in both surviving and nonsurviving groups had decrease tendency (*P* < 0.05) (Figure 2).

## 4. Discussion

Recently, PCT, sTREM-1, CRP, and NT-pro-BNP cytokines were widely used to diagnose sepsis and reflect the severity, but the results were not the same. Meanwhile, there were few studies to put so many biomarkers in one study, particularly how to combine the biomarkers, and clinical severity scores remained unclear.

The present study showed that the serum levels of strem-1 and PCT in nonsurvival group were higher than those in the survival group; meanwhile, they decreased in survival group, but stayed in high levels even increased in the nonsurvival group during sepsis time course. Thus, all these data indicated that strem-1 and PCT could predict sepsis prognosis. Many previous studies have shown that dynamic changes in sTREM-1 levels could predict survival and mortality of patients at the early stage of sepsis [10, 11]. sTREM-1 is widely used to diagnose sepsis [7, 12]. In the present study, serum strem-1 levels of patients in the nonsurvival group were significantly higher than those in the survival group on day 1; it decreased in survival group, but it even increased in the nonsurvival group. All these data indicated that strem-1 could serve as an indicator for sepsis prognosis. Some studies failed to find the association between strem-1 and poor outcome [8, 13]. At a cutoff of 252.05 pg/mL, strem-1 measurements yielded a sensitivity of 85.7%, specificity of 75.7%, positive predictive value of 70.6%, negative predictive value of 88.2%, and an accuracy of 79.4% for differentiating nonsurvivors from survivors. PCT is normally produced in the C cells of the thyroid gland; plasma PCT levels in healthy humans are approximately 5–50 pg/mL in normal state; its half-time is about 22–33 hours in serum. Many tissues and cells except thyroid gland produce and release that PCT on systemic inflammation [14]. Several previous studies reported PCT could serve as a useful tool to distinguish sepsis from systemic inflammatory response syndrome [15, 16]. On the other hand, PCT could reflect the severity of sepsis and outcome. A study by Christophe Clec'h and coworkers found that serum PCT on day 1 was significantly higher in patients with than without septic shock. Meanwhile, among patients with sepsis, PCT concentrations were significantly higher in those who died than in the survivors, at all four measurement time points [17]. Similar results were drawn from other investigations [16, 18]. Very few studies failed to find the prognostic value [19]. At a cutoff of 10.64 ng/mL, procalcitonin measurements yielded a sensitivity of 76.2%, specificity of 81.7%, positive predictive value of 53.5%, negative predictive value of 67.8%, and an accuracy of 61.8% for differentiating nonsurvivors from survivors.

NT-pro-BNP has been found to be a useful markers in the diagnosis, management, and prognosis of patients with congestive heart failure and was secreted into blood in response to atrial or ventricular wall stretch. The half-life is 1-2 hours. It has been used to predict the sepsis outcome recently. A recent meta-analysis suggested that an elevated NT-pro-BNP level may prove to be a powerful predictor of mortality in septic patients [20]. In our study, there was no difference in NT-pro-BNP level between groups on day 1, but the NT-pro-BNP levels in the nonsurvival group were higher than those in the survival group on days 3 and 5. Meanwhile, serum NT-pro-BNP level showed a decreased trend in the survival group, but there was no decrease tendency in the nonsurvival group. We concluded that NT-pro-BNP may predict sepsis 28-day mortality in different stages. One research demonstrated that elevated serum NT-pro-BNP value represented an independent predictor for poor ICU outcome in the presence of clinical severity scores; the cut-off in admission NT-pro-BNP that best predicted outcome was 941 pg/mL [21].

CRP is an acute phase protein and a sensitive systemic marker of inflammation and tissue damage. The secretion of CRP begins within 4–6 h after stimulus, doubles every 8 h thereafter, and peaks at 36–50 h [22]. The role of CRP in sepsis prognostic value seemed different. In our study, there was no significant difference between survivors and nonsurvivors during the three measures, similar to previous study [23], indicating that CRP was just an inflammatory biomarker and failed in reflecting sepsis severity. Luzzani et al. [24] have reported that CRP levels in severe sepsis were lower than those in sepsis, suggesting that CRP levels did not reflect the severity of sepsis.

IL-6 and IL-10 are important proinflammatory and anti-inflammatory cytokines in sepsis. In our study, serum IL-6 levels of patients in the nonsurvival group were significantly higher than those in the survival group on days 1, 3, and 5. There was no difference in IL-10 levels between the groups. Serum IL-6 level showed a decrease trend in the survival group. There were no statistical dynamic changes in IL-10 levels in the two groups. The above results showed that IL-6 had the prognostic utility for sepsis, whereas IL-10 did not show the power. Previous researches demonstrated conflicting results. Suárez-Santamarí and coworkers [25] enrolled 253 hospitalized septic patients; they found that IL-10 and IL-6 were the best predictors, whereas PCT showed only moderate predictive value for mortality. Another study investigated the prognostic value of IL-6, PCT, and CRP in critically ill patients during the first increase of fever; only IL-6 levels were significantly higher in nonsurvivors compared with survivors, in which prognostic value was superior to PCT and CRP [26]. In contrast, Kawczyński and Polakowska [27] indicated that the predictive value of IL-10 plasma concentration was better than that of IL-6.

To sum up, sTREM-1, PCT, and IL-6 serum values attribute to the prognosis of sepsis during the time course. The dynamic changes of biomarkers and cytokines were more meaningful for predicting the sepsis procession. The higher the serum values were, the poorer the outcome was. Schneider and coworkers [28] retrospectively analyzed the relationships between serum PCT, IL-6, and APACHEII score and prognosis of 220 patients on the first day after operation. They found that PCT was the sole independent predictor of 28-day mortality, in which prognostic ability was superior to those of IL-6 and APACHEII score. Zhang et al. [11] suggested that serum sTREM-1 levels reflected the severity of sepsis more accurately than those of PCT and CRP and were more sensitive for dynamic evaluations of sepsis prognosis. Facing the results, we wonder which was the best predictor and how to combine them together and which was more valuable compared to clinical severity scores. APACHE II and SOFA scores have been widely used to validate mortality risk stratification. In our study, we used ROC and logistic regression model to search for the best predictor. Based on ROC analysis, sTREM-1 and PCT showed the equal prognostic ability (0.792 for PCT, 0.862 for sTREM-1, *P* = 0.291), whereas their prognostic utility was inferior to that of APACHEII and SOFA scores which had equal power to predict outcome (0.923 for APACHEII score, 0.953 for SOFA score, *P* = 0.375). Logistic regression model showed that serum sTREM-1, PCT, and SOFA score were the independent predictors of 28-day mortality, which was supported by other result [17].

Our prospective investigation has certain advantages in relation to previous studies. As far as we know, the interrelationship between sTREM-1, PCT, NT-pro-BNP, cytokines, and clinical severity scores for mortality prediction in general ICU patients has not been previously evaluated. Our research firstly discovered that sTREM-1 and PCT had the equal prognostic ability for sepsis mortality and were superior to other parameters. The prognostic difference may be dependent on their biologic and kinetics characteristics. Previous study has indicated that the iteraction of TREM-1 and interact adaptor protein DAP12 can stimulate neutrophil and monocyte-mediated inflammatory response via the triggering and release of pro-inflammatory cytokines and chemokines. sTREM-1 increases quickly when exposued to infection, and its half-time is short. In bacterial infections, serum PCT levels start to rise at 4 h after the onset of systemic infection and peak at between 8 and 24 h; it decreased 50% every 24 hours along with therapy. In contrast, CRP rises slowly and peaks 36 h after an endotoxin challenge. The mechanism of NT-pro-BNP release in sepsis is complex, and kinetics characteristic is unknown. IL-6 and IL-10 rise quickly and peak at 2–4 hours and maintain a short time. The patients admitted to ICU often delayed more than 24 hours, either CRP or cytokines serum concentration was unable to reach the peak at the period of sepsis. Of course, the exact roles of biomarkers and cytokines in sepsis process are not clear, and need to be further studied.

Although we tried our strength, there were several limitations in the present study. Firstly, our study chosen a part of sepsis biomarkers and did not put all biomarkers in the research. The number of univariate factor with difference will influence the logistic analysis results. Of course, it was a costly and unnecessary task to do so. Secondly, every biomarker has its own dynamic characteristics; meanwhile the patients were not in the same sepsis stages in the study; thus the explanation for the results would be influenced. Thirdly, we excluded patients with previous heart diseases history, but we did not perform the UCG to evaluate cardiac function. The conclusion we drawn would influence the explanation for NT-pro-BNP. Fourthly, the observed periods were not long enough. Finally, the sample size of the study was small and larger studies are needed.

## 5. Conclusions

In summary, elevated serum sTREM-1 and PCT levels provide superior prognostic accuracy to other biomarkers. Combination of serum sTREM-1 and PCT levels and SOFA score can offer the best powerful prognostic for sepsis mortality. In the future, in order to improve the accuracy of the prognosis of sepsis, the combination of novel biomarkers and traditional markers of sepsis, reflecting different aspects of sepsis, is an attractive advice and is worthy of further investigation [29].

## Figures and Tables

**Table 1:** Clinical characteristics of patients on admission to ICU.

Parameters	Survivors (*n* = 60)	Nonsurvivors (*n* = 42)	*P* value
Age (years)	64.63 ± 21.35	60.36 ± 19.68	0.307
Sex (male/female)	35/25	14/28	0.692
Initial sites of infection			
Lung (%)	36 (60.0)	23 (54.8)	0.091
Urinary tract (%)	7 (11.7)	8 (19.0)	0.096
Gastrointestinal (%)	5 (8.3)	3 (7.1)	0.480
Blood infection (%)	6 (10.0)	2 (4.8)	0.157
Skin and soft tissue (%)	1 (1.7)	3 (7.1)	0.317
Others	4 (6.7)	3 (7.1)	0.705
Organism			
G^+^ bacterium (%)	14 (23.3)	8 (19.0)	0.201
G^−^ bacterium (%)	16 (26.7)	12 (28.6)	0.450
Fungi (%)	6 (10.0)	2 (4.8)	0.157
Mixed (%)	10 (16.7)	4 (9.5)	0.109
None detected (%)	14 (23.3)	16 (38.0)	0.715
APACHE II score	13.15 ± 6.21	27.38 ± 7.53	0.000
SOFA score	2.82 ± 2.42	10.33 ± 4.14	0.000

Data are expressed as mean ± SD or number (percentage). APACHE II: acute physiology and chronic health evaluation; SOFA: sequential organ failure assessment.

**Table 2:** Comparison of serum biomarkers and cytokines levels on day 1.

Parameters	Survivors (*n* = 60)	Nonsurvivors (*n* = 42)	*P* value
PCT (ng/mL)	2.63 (1.14, 10.00)	11.95 (10.97, 52.00)	0.000
sTREM-1 (pg/mL)	161.95 (124.25, 260.68)	320 (287.60, 418.42)	0.000
NT-pro-BNP (pg/mL)	360.4 (178.15, 1204.5)	539 (314.5, 785.4)	0.198
CRP (mg/dL)	6.82 (4.25, 13.70)	7.24 (6.46, 10.30)	0.612
IL-6 (ng/L)	18.49 (10.52, 21)	31.92 (14.90, 83.94)	0.000
IL-10 (ng/L)	105.59 (96.16, 182.46)	105.77 (76.92, 261.06)	0.488

Data are expressed as median (interquartile ranges). PCT: procalcitonin; sTREM-1: soluble triggering receptor expressed on myeloid cells-1; NT-pro-BNP: N-terminal probrain natriuretic peptide; CRP: C-reactive protein; IL-6: interleukin-6; IL-10: interleukin-10.

**Table 3:** Univariate predictors of 28-day mortality on admission based on ROC.

Parameters	AUC	95% CI	*P *	Threshold	Sensitivity (%)	Specificity (%)	PPV (%)	NPV (%)	Accuracy (%)
PCT (ng/mL)	0.792	0.697–0.887	0.000	10.65	76.2	81.7	53.5	67.8	61.8
sTREM-1 (pg/mL)	0.856	0.784–0.929	0.000	252.05	85.7	75.7	70.6	88.2	79.4
NT-pro-BNP (pg/mL)	0.575	0.463–0.688	0.198	264	81	60	48.6	75	56.9
CRP (mg/dL)	0.53	0.414–0.645	0.612	6.445	81	48.3	52.3	78.4	61.8
IL-6 (ng/L)	0.731	0.635–0.828	0.000	12.66	88.1	46.7	53.6	84.4	63.7
IL-10 (ng/L)	0.54	0.42–0.66	0.49	112.98	50	53.3	42.9	60.4	52.0
APACHE II	0.923	0.869–0.977	0.000	23.5	83.3	95	92.1	95	90.2
SOFA	0.953	0.917–0.907	0.000	6.5	88.1	95	86.8	85.9	86.3

**Table 4:** Multiple logistic regression analysis of variables for predicting 28-day mortality.

Variables	*P* value	Odds ratio (95% Confidence Interval)
sTREM-1 > 252.5 pg/mL	0.044	1–1.028
PCT > 10.65 ng/mL	0.025	0.894–0.992
SOFA > 6.5	0.000	1.441–3.631

The following variables were entered into the regression model on day 1: PCT, sTREM-1, IL-6, APACHE II and SOFA scores.
